# Role of the gap junctions in the contractile response to agonists in pulmonary artery from two rat models of pulmonary hypertension

**DOI:** 10.1186/1465-9921-12-30

**Published:** 2011-03-17

**Authors:** Marie Billaud, Diana Dahan, Roger Marthan, Jean-Pierre Savineau, Christelle Guibert

**Affiliations:** 1 INSERM, U1045, 146 rue Léo Saignat, F-33076 Bordeaux, France; 2 Université Bordeaux Segalen, Centre de Recherche Cardio-Thoracique de Bordeaux, F-33076 Bordeaux, France; 3 CHU de Bordeaux, F 33076 Bordeaux, France

**Keywords:** pulmonary hypertension, gap junctions, connexin, vasoreactivity, chronic hypoxia, monocrotaline, connexin-mimetic peptides

## Abstract

**Background**

Pulmonary hypertension (PH) is characterized by arterial vascular remodelling and alteration in vascular reactivity. Since gap junctions are formed with proteins named connexins (Cx) and contribute to vasoreactivity, we investigated both expression and role of Cx in the pulmonary arterial vasoreactivity in two rat models of PH.

**Methods**

Intrapulmonary arteries (IPA) were isolated from normoxic rats (N), rats exposed to chronic hypoxia (CH) or treated with monocrotaline (MCT). RT-PCR, Western Blot and immunofluorescent labelling were used to study the Cx expression. The role of Cx in arterial reactivity was assessed by using isometric contraction and specific gap junction blockers. Contractile responses were induced by agonists already known to be involved in PH, namely serotonin, endothelin-1 and phenylephrine.

**Results**

Cx 37, 40 and 43 were expressed in all rat models and Cx43 was increased in CH rats. In IPA from N rats only, the contraction to serotonin was decreased after treatment with ^37-43^Gap27, a specific Cx-mimetic peptide blocker of Cx 37 and 43. The contraction to endothelin-1 was unchanged after incubation with ^40^Gap27 (a specific blocker of Cx 40) or ^37-43^Gap27 in N, CH and MCT rats. In contrast, the contraction to phenylephrine was decreased by ^40^Gap27 or ^37-43^Gap27 in CH and MCT rats. Moreover, the contractile sensitivity to high potassium solutions was increased in CH rats and this hypersensitivity was reversed following ^37-43^Gap27 incubation.

**Conclusion**

Altogether, Cx 37, 40 and 43 are differently expressed and involved in the vasoreactivity to various stimuli in IPA from different rat models. These data may help to understand alterations of pulmonary arterial reactivity observed in PH and to improve the development of innovative therapies according to PH aetiology.

## Background

Gap junctions are clusters of intercellular channels resulting from the connection of two hexameric assembly of membrane proteins termed connexins (Cx) [1]. Each hexameric assembly is also known as a hemichannel or connexon, localized on the membrane of two adjacent cells and arranged with identical Cx (homomeric connexon) or different Cx (heteromeric connexon) with various possible combinations [2]. Such process has functional consequences and provides an efficient cellular strategy to finely regulate cell-to-cell communication. In the vascular wall, the most common connexins are Cx37, Cx40 and Cx43 in endothelial and smooth muscle cells [3]. Gap junctions allow cell-to-cell coupling in between vascular cells of the same type, namely endothelial or smooth muscle cells but they are also present in between endothelial and smooth muscle cells (myoendothelial gap junctions). Gap junctions allow direct diffusion of ions and small molecules between adjacent cells in almost all animal tissues. As a consequence, gap junctions are vital components in the coordination of vascular response and are therefore essential for the control of vascular functions including vasoreactivity and cell proliferation [3]. There is now accumulating evidence indicating that Cx may play a role in a variety of vascular diseases including systemic arterial hypertension [4]. For instance, elevated pressure has been shown to increase the expression of Cx 43 in cultured cells from aorta [5]. However, the role of gap junctions in pulmonary hypertension (PH) remains largely unknown.

PH is a multifactorial disease characterized by a progressive increase in pulmonary vascular resistance caused by vasoconstriction, vascular cell proliferation and obliteration of pulmonary microvasculature. These processes lead to right heart failure and ultimately to death [6]. PH occurs in a variety of clinical situations and is associated with a broad spectrum of histological patterns and molecular abnormalities. Because of this diversity, early diagnosis is difficult and efficient treatments are still lacking. The recent revision of the classification of PH distinguishes five groups [7]. Among these groups, the category 1 PH also known as pulmonary arterial hypertension (PAH) includes idiopathic PAH, familial PAH and acquired PAH, the latter of which being associated with other diseases such as HIV or connective tissue diseases. The non-category 1 PH previously known as secondary PH includes the category 3 which is a widely distributed PH secondary to alveolar hypoxia as a result of lung disease such as chronic obstructive pulmonary disorder (COPD). Although, PH has progressively evolved from a fatal to a chronic disease, none of the currently available therapies is curative [8]. Despite intensive research, PH remains an important medical challenge and a better knowledge of the underlying molecular and cellular mechanisms remains crucial for the development of new or additional innovative therapies.

To comprehensively address the issue of the role of gap junction in PH, we have used two different rat models, the hypoxia and monocrotaline-induced models that share pathophysiological characteristics with category 3 and category 1 PH, respectively. Like category 1 and 3 PH patients, monocrotaline- and the chronic hypoxia-treated rats (MCT and CH rats respectively) exhibit high circulating concentrations of serotonin (5-HT), endothelin-1 (ET-1) and norepinephrine (an adrenoceptor agonist) [9-14]. These increased concentrations of 5-HT, ET-1 and norepinephrine participate to the increase in pulmonary vascular tone [14-16]. Moreover, reduced expression of a variety of potassium channels and mainly voltage-gated and calcium-activated potassium channels leads to membrane depolarisation, voltage-gated calcium channel opening and cytosolic calcium increase in rat and patients with PH [15,17]. The resulting intracellular calcium increase participates to the high pulmonary arterial vascular tone observed in PH.

In the normal pulmonary arterial wall, we have recently demonstrated a functional role of gap junctions in contractile and calcium responses to 5-HT [18]. The aim of the present study was thus to examine the role of gap junctions in the abnormal pulmonary arterial wall in PH. We have demonstrated that Cx 37, 40 and 43 are expressed in intrapulmonary arteries (IPA) from the normoxic (N), chronic hypoxia (CH) and MCT rats and that Cx 43 is overexpressed in CH rats. By using Cx-mimetic peptides as blockers of gap junctions [18,19], we also highlight the role of Cx in the contractile responses to stimuli, already known to be involved in PH, namely 5-HT, ET-1, phenylephrine (an α1-adrenoceptor agonist, Phe) and depolarising solutions (high potassium solutions).

## Methods

### Animal models of PH

Male Wistar rats (300-400 g) were separated into 3 groups: the first group (control or normoxic rats - N rats) was housed in ambient room air, the second group (chronic hypoxic rats - CH rats) was exposed to chronic hypoxia for 3 weeks in a hypobaric chamber (50 kPa) and the third group was injected with a single intraperitoneal dose of monocrotaline (60 mg.kg^-1^) (MCT rats) and used 4 weeks later. MCT (Sigma, St Quentin Fallavier, France) was dissolved in an equal volume of HCl (1 M) and NaOH (1 M). We checked that an injection of an equal volume of isotonic saline solution did not modify the pulmonary arterial reactivity to agonists. All animal care and experimental procedures complied with the recommendations of the Federation of European Laboratory Animals Science Association, and were approved by the local ethics committee (Comité d'éthique régional d'Aquitaine - referenced AP 2/11/2005).

PH was assessed by measuring both the mean pulmonary arterial pressure (mean PAP) and right ventricle hypertrophy. To measure PAP, N, CH and MCT rats were anesthetized with xylazine 10 mg.kg^-1 ^and ketamine 50 mg.kg^-1 ^by intraperitoneal injection and mean PAP was measured, in closed-chest rats, through a catheter inserted in the right jugular vein, then through the right atria and the right ventricle into the pulmonary artery, and attached to a Baxter Uniflow gauge pressure transducer. Pressure was recorded with an automatic monitor (Physiogard SM 785, ODAM, Wissembourg, France) [20]. Right ventricle hypertrophy was estimated by the ratio of right ventricle (RV) to left ventricle plus septum (LV+S) weight (Fulton's index).

### Tissue Preparation

Rats were sacrificed using CO_2 _asphyxia according to the animal care and use local committee (Comité d'éthique régional d'Aquitaine - referenced AP 2/11/2005). The left lung was rapidly removed and rinsed in Krebs solution containing (in mM): 118.4 NaCl, 4.7 KCl, 1.2 MgSO_4_, 25 NaHCO_3_, 1.2 KH_2_PO_4_, 2.5 CaCl_2_, and 11.1 D-glucose, pH 7.4 with NaOH. Intrapulmonary arteries with an external diameter of 300-350 μm (intrapulmonary artery of the third order - IPA3) and 1.5-2 mm (intrapulmonary artery of the first order - IPA1) were then dissected free from surrounding connective tissues under binocular control.

### Quantitative RT-PCR

#### RNA Extraction

IPA from one rat was homogenized using 600 μl of Trizol (Invitrogen, Cergy Pontoise, France), then, 120 μl of chloroform (Sigma, St Quentin Fallavier, France) was added. RNA was extracted from the aqueous phase after centrifugation at 15,000 *g *for 15 min. RNA was precipitated in the presence of isopropanol (Sigma, St Quentin Fallavier, France) at 20°C overnight. Pure RNA was obtained by centrifugation at 15,000 *g *for 15 min and was washed with 80% ethanol (Sigma, St Quentin Fallavier, France). The concentration of RNA was measured spectrophotometrically by GeneQuant RNA/DNA calculator (Amersham Pharmacia, Orsay, France). Total RNA (1 μg) was reverse transcribed into cDNA by using AMV reverse transcriptase (Promega, Charbonnières-les-bains, France), RNase inhibitor, and oligo d(T) as a primer at 42°C for 60 min followed by heating at 94°C for 3 min.

#### Real-time Quantitative Polymerase Chain Reaction (PCR)

Real-time quantitative PCR was performed with a Rotor-Gene 2000 (Corbett Research, Cortaboeuf, France) as previously described [21]. cDNA from 10 ng of total RNA were added to 0.2 μl of 50X Titanium Taq DNA Polymerase combined to its buffer (Clontech Laboratories, Saint Germain-en-Laye, France), 1 mM dNTP, each of the appropriate primer (Sigma Genosys; see table 1 for concentrations and sequences, St Quentin Fallavier, France), and 0.5X SYBR Green (Molecular Probes, Cergy Pontoise, France).

**Table 1:** Sequences of the primer pairs (S: sense; AS: antisense) for housekeeping genes (GAPDH, HPRT, PLRPO and YWHAZ) and genes of interest (Cx 37, Cx 40 and Cx 43) are shown as well as GenBank accession number, product length, product Tm and concentrations

Gene	Sequence	GenBank accession number	Product length (pb)	Product Tm (°C)	Concentration (nM)
GAPDH	S: ATTCTACCCACGGCAAGTT	NM_017008	153	89.4	200
					
	AS: CGCCAGTAGACTCCACGACATA				

HPRT	S: TGTTGGATATGCCCTTGACTA	NM_012583	178	85.6	100
					
	AS: AGATGGCCACAGGACTAGAAC				

PLRPO	S: AGGTGGGAGCCAGCGAAGC	NM_022402	208	91.7	100
					
	AS: GCAACAGTCGGGTAGCCAATC				

YWHAZ	S: AGCCGAGCTGTCTAACGAG	NM_013011	291	88.4	100
					
	AS: GCCAAGTAGCGGTAGTAGTCA				

Cx 37	S: GGTGGCAGAGGACGGTCGTCT	NM_021654	133	85.3	200
					
	AS: CCATGGTCCAGCCGTAGAGA				

Cx 40	S: GGAAAGAGGTGAACGGGAAG	NM_01280	197	91.3	200
					
	AS: GGGCCTCGAGACATAACAGTT				

Cx 43	S:TCTGCCTTTCGCTGTAACACT	NM_012567	117	87.5	200
					
	AS: GGGCACAGACACGAATATGAT				

PCR conditions used were identical to those used previously [18]. PCR negative controls were systematically made by using water instead of cDNA. All specific primers were designed by using the primer analysis software (Oligo 6.6, Molecular Biology Insights, Cascade, USA). The efficiency of PCR reactions was always more than 90%. Specificity of the amplified PCR products was checked with melting curve analysis and by electrophoresis analysis on a 2% agarose gel containing SYBR Green.

### Western Blot

IPA3 from 4 rats were homogenized and proteins were extracted as previously described [22]. Such protein extraction from IPA3 was performed from 4 different pools of 4 rats each. Protein extracts were subjected to electrophoresis on a 10% acrylamid reducing gel, and transferred to polyvinylidene fluoride (PVDF) membranes (Immobilon-P, Millipore, Molsheim, France). The immunoblots were then incubated using rabbit anti-Cx 37, rabbit anti-Cx 40 or mouse anti-Cx 43 (Zymed, Paris, France) overnight at 4°C. After incubation with appropriate secondary antibodies coupled to horseradish peroxydase (HRP, Santa Cruz, Heidelberg, Germany) for 2 h at room temperature, immunoblots were then revealed by enhanced chemiluminescence acquired using Kodak Image Station 4000 MM. Band densities were quantified using GeneTool software (SynGene, Cambridge, United Kingdom). Immunoblots were then stripped and revealed with mouse anti β-actin.

### Immunofluorescence

Immunolabelling of IPA3 isolated from N, MCT and CH rats was performed as previously described [18]. IPA3 were fixed and cut in 10 μm sections with a cryostat. Sections were first incubated in blocking buffer containing 0.1% Triton-X100 (Sigma, St Quentin Fallavier, France) and 1% bovine serum albumin (BSA) in PBS for 1 h at room temperature and then in the primary antibodies (same antibodies as the ones used for Western Blot) overnight at 4°C. Sections were then incubated with the appropriate secondary antibodies coupled to Alexa 546 (Molecular Probes, Cergy Pontoise, France). Nuclei were labeled with 45 μM Hoechst 33342 (Molecular Probes, Cergy Pontoise, France). Sections were then observed with a laser scanning confocal microscope TE2000 (Nikon, Champigny-Sur-Marne, France) with a × 60, 1.40 NA plan apochromat oil-immersion objective. Excitation was obtained with a diode laser at 408 nm to observe nuclei, an argon laser at 488 nm to observe autofluorescence of the internal and external elastic lamina and a helium-neon laser at 543 nm to observe Cx labelling. The emitted light was filtered as appropriate: 450 ± 35 nm for nuclei (blue), 515 ± 30 nm for internal elastic lamina (green), and 605 ± 75 nm for Cx labelling (red).

### Isometric tension measurements

IPA1 were divided into short tubular segments with an external diameter of 1.5-2 mm and used for isometric contraction measurement as reported previously [23]. Arterial rings were mounted in isolated organ bath systems, containing Krebs solution at 37°C and bubbled continuously with either 15% O_2_, 5% CO_2 _for IPA1 isolated from N and MCT rats, or with 9% O_2_, 5% CO_2 _for IPA1 isolated from CH rats. As previously determined [22], an initial load of 0.8 g was applied to arterial rings isolated from control rats, and 1.6 g to arterial rings isolated from CH and MCT rats. Tissues were allowed to equilibrate for 1 h in Krebs solution and washed out every 15 min. A high KCl solution (80 mM) was applied in order to obtain a reference contraction used to normalize subsequent contractile responses. 80 mM high KCl solution induced similar contractions in pulmonary arterial rings from the three rat models (1036 ± 50 mg, n = 67; 1205 ± 143 mg, n = 64; 953 ± 54 mg, n = 68 in N, CH and MCT rats respectively; *P *> 0.05). Contractile responses to different agonists were then tested by constructing a cumulative concentration-response curve (CCRC) to 5-HT (10 nM to 100 μM), ET-1 (0.1 to 100 nM), Phe (0.1 nM to 10 μM) or high KCl solutions (4.7 to 100 mM). We inhibited gap junction communications through Cx 37 and Cx 43 or Cx 40 with 300 μM synthetic connexin-mimetic peptides: ^37-43^Gap 27 (SRPTEKTIFII; Peptide 2.0 Inc, Shirley, USA), ^40^Gap 27 (SRPTEKNVFIV; Genscript, Piscataway, USA) respectively [18,19]. When indicated, peptides were preincubated during 1 h, and then CRC to agonist was performed in the presence of the peptide. Specificity of these peptides was checked in a previous study [18]. High potassium solutions were obtained by substituting an equimolar amount of KCl for NaCl from Kreb's solution. To determine the role of the gap junctions in conditions close to physiological conditions, we studied the vessels with intact endothelium. Endothelial function was tested on each ring by examining the relaxation induced by 10 μM carbamylcholine on 0.3 μM Phe-induced preconstricted pulmonary arterial rings. Passive and active mechanical properties were assessed using transducer systems, coupled to IOX software (EMKA technologies, Paris, France) in order to facilitate data acquisition and analysis [23].

### Data analysis and statistics

Results are expressed as mean ± SEM; n indicates the number of rats for quantitative RT-PCR, Fulton ratio and mean PAP whereas it indicates the number of experiments from 4 pools of rats (4 rats per pool) for Western Blot. For isometric tension measurements, n indicates the number of pulmonary arterial rings. CCRC to agonists were fitted using Origin 6 software (Originlab, Paris, France) to the logistic equation: T = ((T0-Tmax)/(1+(X/EC_50_)p)) + Tmax where T, Tmax and T0 are, respectively, the amplitude of tension developed, the relative maximum and minimal tensions for a given agonist concentration normalized to the 80 mM KCl responses, X is the concentration of agonist used, EC_50 _is the concentration of agonist which produces half maximal tension, and p is the slope of the curve. Global comparisons of the CCRC were performed using ANOVA. When global comparisons of the curves were significantly different, unpaired Student's t tests were performed on maximal effect (Emax) and/or EC_50_. EC_50 _was determined only when the maximal contraction was achieved. Quantitative RT-PCR results were analyzed with the GeNorm method [18]. Statistical analyses were performed on all other data using a non parametric test for unpaired samples (Mann-Whitney test). A value of *P *< 0.05 was considered significant.

## Results

### Assessment of pulmonary hypertension in two rat models

The mean PAP value was significantly increased from 15 ± 2 mmHg in N rats (n = 5) to 30.4 ± 1.03 mmHg in CH rats (n = 5) or 29.8 ± 1.3 mmHg in MCT rats (n = 5). The Fulton's index was also significantly increased in each pulmonary hypertensive rat model indicating a right ventricle hypertrophy in CH and MCT rats compared to N rats (0.63 ± 0.04, n = 20; 0.54 ± 0.03, n = 20 and 0.27 ± 0.03, n = 20 respectively).

To differentiate MCT from CH rat models, we examined the endothelial NO-dependent function by inducing a relaxation with 10 μM carbamylcholine on 0.3 μM Phe-induced preconstricted pulmonary arterial rings. Relaxation to carbamylcholine 10 μM was not significantly changed in CH compared to N rats whereas it was almost abolished in MCT rats (58.1 ± 3.0% and 3.3 ± 0.9% of the precontraction to 0.3 μM Phe in CH and MCT rats respectively, n = 48 and 44 *vs *64.1 ± 2.9% in N rats, n = 48).

### Cx 37, 40 and 43 are expressed in the pulmonary arterial wall from N, CH and MCT rats

**Figure 1:**
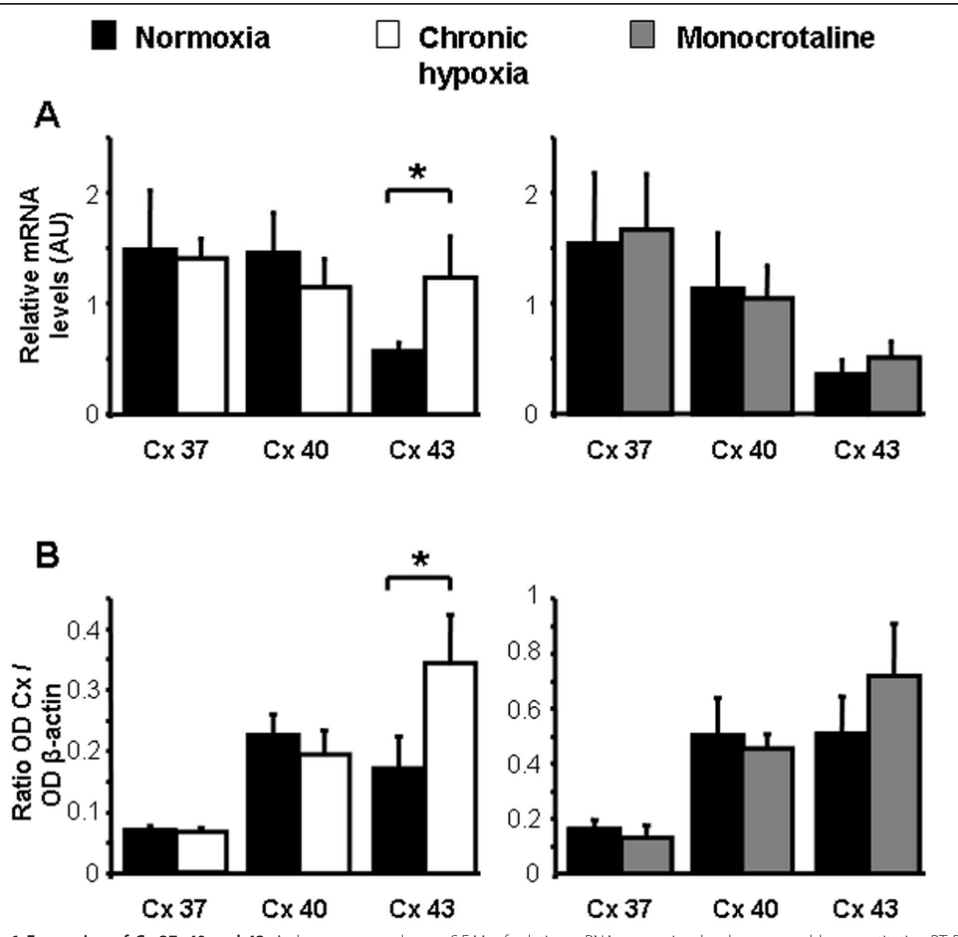
**Expression of Cx 37, 40 and 43**. A shows mean values ± S.E.M. of relative mRNA expression levels measured by quantitative RT-PCR in pulmonary arteries from N rats (normoxia, black column, n = 4 - 5), chronic hypoxic rats (chronic hypoxia, white column, n = 9) and monocrotaline-injected rats (monocrotaline, grey column, n = 5 - 7). B shows protein expression measured by Western Blot and normalized to β actin expression level in normoxic rats (black column, n = 6 - 12), chronic hypoxic rats (white column, n = 9 - 12) and monocrotaline-injected rats (grey column, n = 5 - 7). * means a significant difference for *P *< 0.05.

Quantitative RT-PCR and Western Blot experiments have demonstrated the presence of the mRNA and proteins, respectively for Cx 37, 40 and 43 in IPA3 from N, CH and MCT rats (Figure 1A and 1B respectively). Interestingly, a significant increase in both mRNA and protein levels was observed for the Cx 43 in IPA3 from CH rats compared to IPA3 from N rats (Figure 1A and 1B, left panels).

**Figure 2:**
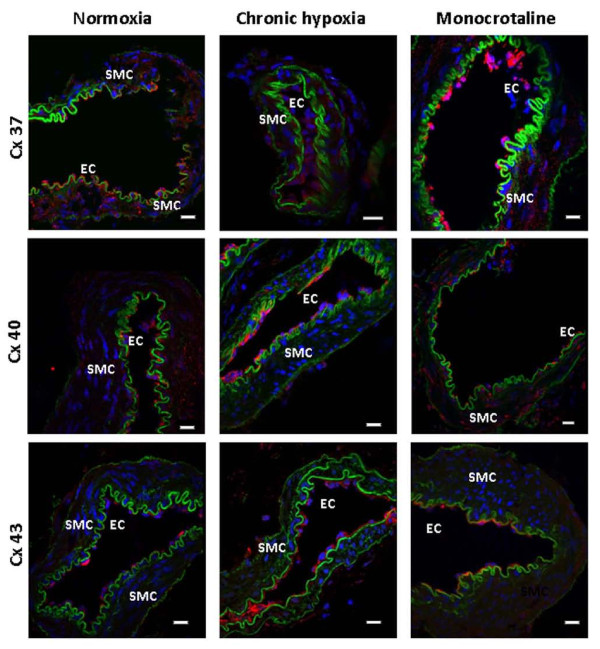
**Localization of Cx 37, 40 and 43 in the pulmonary arterial wall**. Immunofluorescent labelling experiments shows the presence of Cx (red) 37 (top row), 40 (middle row) and 43 (bottom row) in transverse sections of pulmonary arteries from normoxic (normoxia, left column), chronic hypoxic (chronic hypoxia, middle column) and monocrotaline-injected rats (monocrotaline, right column). Green shows the autofluorescent signal of the vessels and blue shows the nuclei. SMC means smooth muscle cells and EC means endothelial cells. 7-8 normoxic rats have been used and the number of IPA3 sections, used for the labelling of Cx 37, 40 and 43, was 21, 22 and 14 respectively. 4-5 monocrotaline-injected rats have been used and the number of IPA3 sections, used for the labelling of Cx 37, 40 and 43, was 11, 17 and 14 respectively. Finally, we have used 3 chronic hypoxic rats and the number of IPA3 sections used for the labelling of the Cx 37, 40 and 43, was 4, 5 and 7 respectively. Scale bars are 15 μm.

Immunofluorescent labelling studies confirmed the presence of the Cx 37, 40 and 43 proteins (Figure 2). The autofluorescence of the external and internal elastic lamina in green allowed us to delimit the smooth muscle layers from the endothelial cells and the adventitia as previously observed [18]. The three connexins were mainly present in the endothelial and possibly in the myoendothelial junctions (Figure 2). Some punctuate labelling, characteristic of the Cx staining, was also sparsely present for Cx 37 and 40 in the smooth muscle of IPA3 from N and MCT rats (Figure 2). Since identical antibodies were used for Western Blot and immunofluorescent labelling experiments, the typical punctuate labelling observed demonstrate the specificity of these Cx antibodies and strengthen our Western Blot results.

### Comparison of the pulmonary arterial reactivity to various stimuli in N, CH and MCT rats

**Figure 3:**
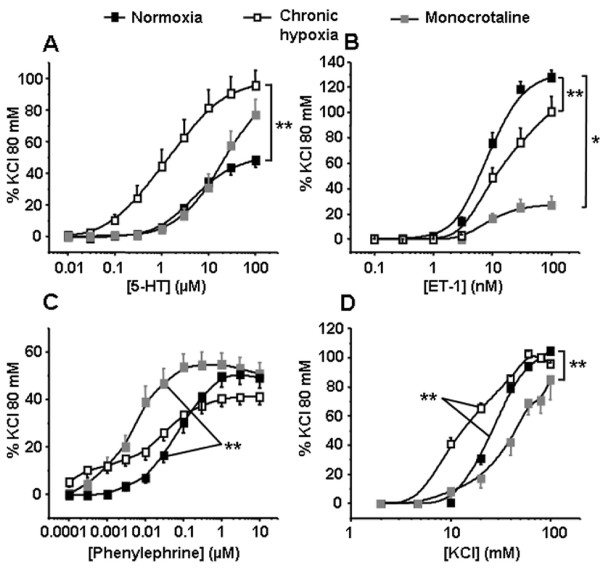
**Contraction induced by 5-HT, ET-1, Phe and high potassium solutions (KCl)**. Isometric tension measurements were recorded on intrapulmonary arteries in response to cumulative concentrations of 5-HT (A, n = 19 - 31), ET-1 (B, n = 17 - 26), Phe (C, n = 21 - 29) and KCl (D, n = 10 - 20) in normoxic, chronic hypoxic and monocrotaline-injected rats (black, white and grey squares respectively). Data are means ± SEM and are expressed as a percentage of the high potassium solution (80 mM)-induced response. ** means a significant difference for *P *< 0.01.

We then compared contractile responses to receptor-dependent or -independent stimuli such as (1) 5-HT, (2) ET-1, (3) Phe, an agonist of the α1-adrenoceptor and (4) a depolarising agent such as high potassium solutions in IPA from N, CH and MCT rats. The contractile responses to 5-HT and high potassium solutions were significantly increased in CH rats (Figure 3A and 3D) whereas those to ET-1 were significantly decreased in both pulmonary hypertensive rat models (CH and MCT rats, Figure 3B). Finally, contractile responses to Phe were significantly increased in MCT rats (Figure 3C).

### Role of Cx 37, 40 and 43 in the reactivity to various stimuli in intrapulmonary arteries from N, CH and MCT rats

Since, (1) Cx 37, 40 and 43 are all expressed (Figure 1 and 2) and (2) 5-HT, ET-1, Phe and depolarisation induce a mechanical activity (Figure 3) in intrapulmonary arteries from CH and MCT rats, we systematically addressed the role of Cx 37, 40 and 43 in the isometric contraction to 5-HT, ET-1, Phe and high potassium solutions by using specific blockers of Cx 40 or Cx 37 and 43 (namely ^40^Gap27 and ^37-43^Gap27 respectively, two Cx-mimetic peptides) in IPA from N, CH and MCT rats.

**Figure 4:**
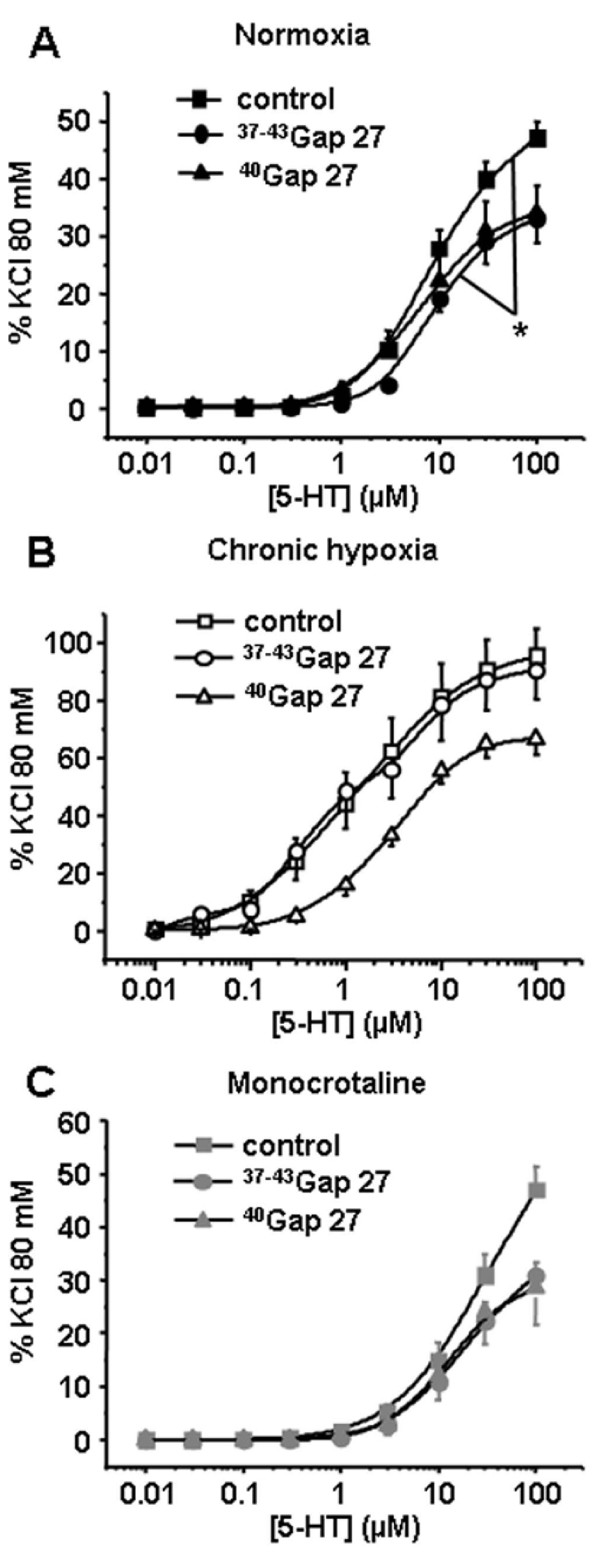
**Contribution of Cx 37, 40 and 43 in the contractile response to 5-HT**. Concentration-response curves to 5-HT were performed in the absence (control, squares) or in the presence of connexin-mimetic peptides targeted against Cx 37 and 43 (^37-43^Gap27, circles) and 40 (^40^Gap27, triangles). Each set of experiments was performed on intrapulmonary arteries from normoxic (normoxia, panel A, black symbols, n = 12 - 24), chronic hypoxic (chronic hypoxia, panel B, white symbols, n = 8 - 19) and monocrotaline-injected rats (monocrotaline, panel C, grey symbols, n = 9 - 20). Data are means ± SEM and are expressed as a percentage of the high potassium solution (80 mM)-induced response. * means a significant difference for *P *< 0.05.

**Figure 5:**
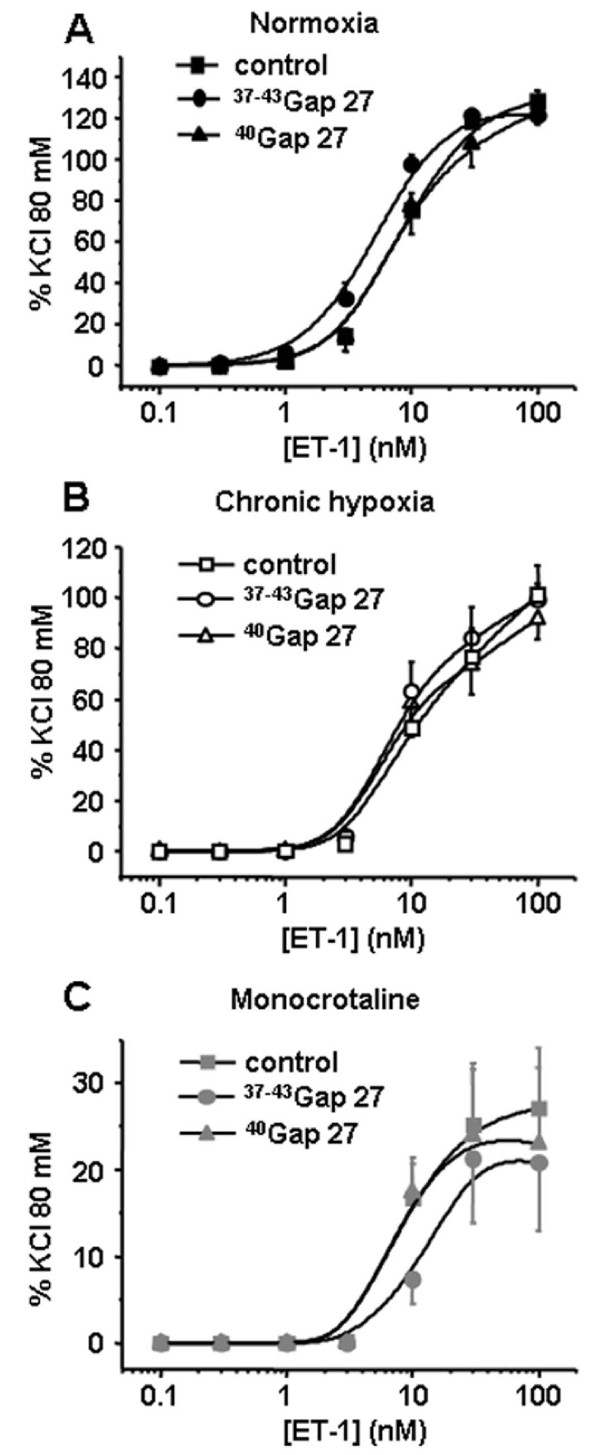
**Contribution of Cx 37, 40 and 43 in the contractile response to ET-1**. Concentration-response curves to ET-1 were performed in the absence (control, squares) or in the presence of connexin-mimetic peptides targeted against Cx 37 and 43 (^37-43^Gap27, circles) and 40 (^40^Gap27, triangles). Each set of experiments was performed on intrapulmonary arteries from normoxic (normoxia, panel A, black symbols, n = 10 - 26), chronic hypoxic (chronic hypoxia, panel B, white symbols, n = 8 - 17) and monocrotaline-injected rats (monocrotaline, panel C, grey symbols, n = 8 - 20). Data are means ± SEM and are expressed as a percentage of the high potassium solution (80 mM)-induced response.

**Figure 6:**
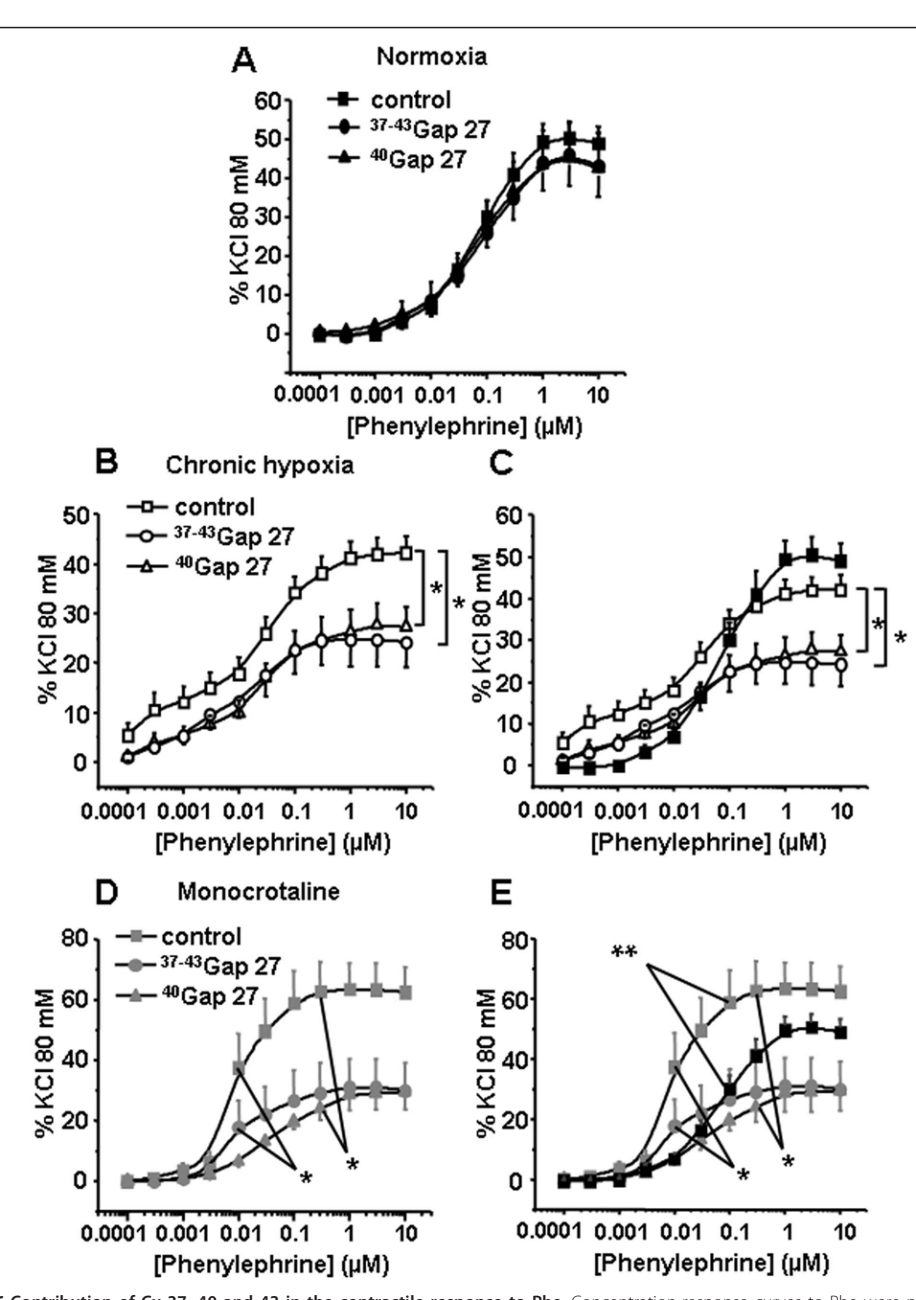
**Contribution of Cx 37, 40 and 43 in the contractile response to Phe**. Concentration-response curves to Phe were performed in the absence (control, squares) or in the presence of connexin-mimetic peptides targeted against Cx 37 and 43 (^37-43^Gap27, circles) and 40 (^40^Gap27, triangles). Each set of experiments was performed on intrapulmonary arteries from normoxic (normoxia, panel A, black symbols, n = 11 - 21), chronic hypoxic (chronic hypoxia, panel B, white symbols, n = 14 - 23) and monocrotaline-injected rats (monocrotaline, panel D, grey symbols, n = 7 - 13). Panels C and E summarize the effects of the Gap27 on the contractile changes to Phe from N to CH or from normoxic to MCT rat models respectively. Data are means ± SEM and are expressed as a percentage of the high potassium solution (80 mM)-induced response. * means a significant difference for *P *< 0.05.

Contractile responses to 5-HT were significantly decreased by 300 μM ^37-43^Gap27 only in IPA from N rats (Figure 4A). In IPA from either rat model, the contractile responses to ET-1 were unchanged following one hour incubation with 300 μM ^37-43^Gap27 or ^40^Gap27 (Figure 5). Interestingly, and inversely to 5-HT, contractile responses to Phe were decreased following 300 μM ^37-43^Gap27 or ^40^Gap27 treatment in IPA from CH and MCT rats (Figure 6 B, C and 6D, E respectively). In N rats, contractile responses to Phe were unchanged by ^37-43^Gap27 or ^40^Gap27 incubation (Figure 6A). The effects of the ^37-43^Gap27 and ^40^Gap27 on contractile changes from N to CH rats and from N to MCT rats are demonstrated in figure 6C and 6E respectively.

**Figure 7:**
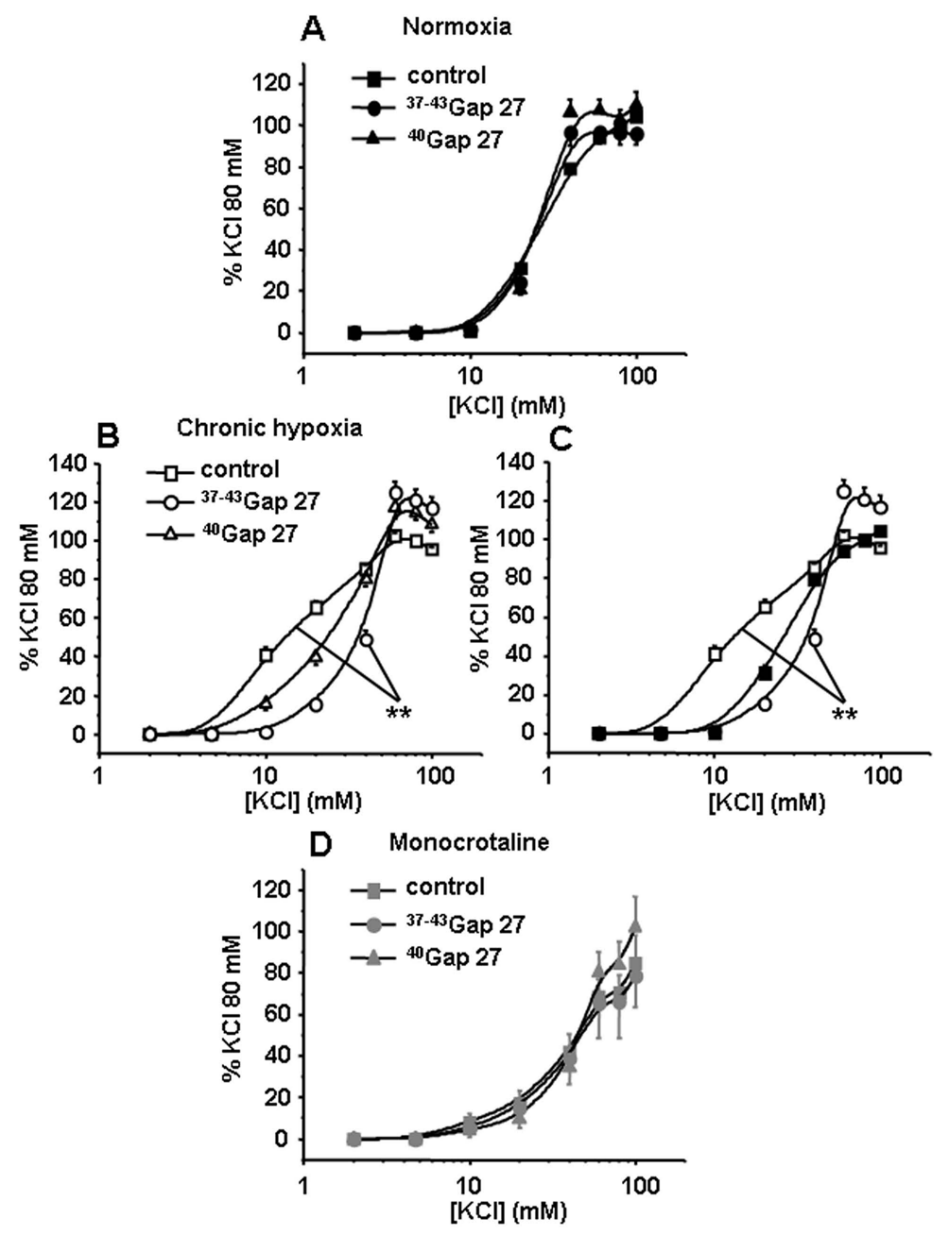
**Contribution of Cx 37, 40 and 43 in the contractile response to high potassium solution (KCl)**. Concentration-response curves to KCl were performed in the absence (control, squares) or in the presence of connexin-mimetic peptides targeted against Cx 37 and 43 (^37-43^Gap27, circles) and 40 (^40^Gap27, triangles). Each set of experiments was performed on intrapulmonary arteries from normoxic (normoxia, panel A, black symbols, n = 8 - 20), chronic hypoxic (chronic hypoxia, panel B, white symbols, n = 8 - 20) and monocrotaline-injected rats (monocrotaline, panel D, grey symbols, n = 4 - 12). Panels C summarizes the effect of the ^37-43^Gap27 on the contractile changes to KCl from normoxic to CH rat model. Data are means ± SEM and are expressed as a percentage of the high potassium solution (80 mM)-induced response. ** means a significant difference for *P *< 0.01.

When using a receptor-independent depolarising agent (high potassium solutions), contractile responses were significantly decreased in the presence of 300 μM ^37-43^Gap27 only in IPA from CH rats (Figure 7B). EC_50 _was significantly increased in the presence of ^37-43^Gap27 in IPA from CH rats (40.2 ± 0.5 mM *vs *15 ± 1.1 mM in the absence of ^37-43^Gap27) indicating a decrease in the sensitivity to high potassium solutions. 300 μM ^40^Gap27 had no effect, under identical conditions, in any of the hypertensive rat models used (Figure 7B and 7D). In the presence of the ^37-43^Gap27, the contraction to KCl in CH rats was identical to the contraction to KCl in N rats (Figure 7C).

## Discussion

Underlying cellular mechanisms as well as targets, governing the pathogenesis of PH, still remain to be fully elucidated. This may be due, at least in part, to the fact that the pathogenesis of PH differs depending on the type of aetiology of this vascular disorder. For this reason, we conducted the present study in two different PH models: MCT induced PH (non hypoxic model) and CH-induced PH (hypoxic model).

Two major excitation-contraction coupling mechanisms are involved in regulating pulmonary vascular tone: electromechanical and pharmacomechanical coupling. We thus adressed agonist-mediated pulmonary contraction (pharmacomechanical coupling induced by either 5-HT, ET-1 or Phe) and depolarisation-mediated contraction (electromechanical coupling induced by high potassium solution). In the present study, IPA from CH rats exhibited hyperreactivity to 5-HT (Figure 3A), which is consistent with previous studies [22,24,25]. Regarding ET-1, vasoconstriction to this agent was only slightly decreased in hypoxic PH whereas it was dramatically decreased in MCT-induced PH. In CH rats, pulmonary arterial contractile responses to ET-1 have been shown to be increased in resistant IPA but decreased in extra pulmonary arteries [16]. Consequently, the slight decrease in the contraction to ET-1 observed in CH rats may be explained by the intermediate size of the pulmonary artery (PA) used for the present contractile experiments (namely PA of the first order or IPA1). In MCT rats, a recent study has shown that although the contraction to ET-1 was not altered, ET-B receptors were decreased in resistance PA [26]. Since the contraction to ET-1 is linked to ET-A receptors in extra-PA and to ET-B receptors in IPA [26,27], the present decreased ET-1 contraction may result from the reported decrease in ET-B receptors in MCT rats. Regarding Phe and in contrast to ET-1, we found that Phe-induced contraction was increased in PA from MCT rats. A recent report from Mam *et al. *(2010) showed a reduced contraction to Phe in CH and MCT rats [28]. This difference may be explained by different animal strain (Sprague Dawley *vs *Wistar rats) and/or different vascular preparation (extra-PA *vs *IPA) and/or different experimental conditions (normobaric *vs *hypobaric hypoxia). Finally, regarding the receptor independent depolarizing agent KCl, pulmonary vascular reactivity to membrane depolarization was increased in CH rats, whereas it was reduced in MCT rats. These results are consistent with previous findings from our laboratory indicating that PA smooth muscle cells (PASMC) from CH rats exhibit a higher resting membrane potential and a higher basal intracellular calcium concentration [22,29]. Moreover, the decreased expression of potassium channels previously observed in CH rats [17] could also contribute to increase the sensitivity to depolarisation.

We have previously shown that Cx 37, 40 and 43, three gap junction proteins commonly found in the vasculature, are expressed in rat PA endothelial cells, while Cx 37 and 40 only are found in PASMC [18]. In the current study, Cx 37, 40 and 43 were also expressed in pulmonary arterial wall from both hypoxic and non-hypoxic PH rats. However, PASMC from CH rats no longer expressed Cx 37 and 40 isoforms, whereas they were sparsely expressed in MCT rats (Figure 2). Interestingly, Cx 43 expression was upregulated in CH rats but not in MCT rats (Figure 1). We cannot exclude that Cx 43 is expressed at the myoendothelial junctions and possibly on the smooth muscle side of these heterocellular structures. Cowan et al. have indeed demonstrated that hypoxia (2.2% O_2 _during 6 h) increased Cx 43 expression in cultured smooth muscle cells from rat thoracic aorta [5]. Consequently, we can hypothesize that the increase in Cx 43 in the IPA from CH rat could be due to the effect of hypoxia on Cx 43 localized on the smooth muscle side of the myoendothelial gap junctions. Interestingly, we have previously demonstrated that Cx 43 present between PASMC and endothelial cells plays an important role in the vasoreactivity to 5-HT in IPA from N rats [18].

Since (i) the three Cx 37, 40 and 43 are expressed in CH and MCT rats (Figure 1 and 2) and (ii) Cx 37 and 43 are involved in the contractile and calcium responses to 5-HT in IPA from N rats [18], we addressed the role of these Cx in the contractile response to various agonists known to contribute to PH (5-HT, ET-1 and Phe) in IPA from CH and MCT rats. In the present study, the effect of Gap27 varied according to the agonists used and the rat model considered. Since we have previously demonstrated that the role of the gap junctions in the contraction depended on the amount of superoxide anion (O_2_^●^) level in smooth muscle, it can be hypothesized that the modulation of O_2_^●^level in IPA from hypertensive rat models may differ according to the agonist. In this respect, we can suggest that, unlike 5-HT, Phe increases O_2_^●^levels in pulmonary hypertensive models and not in N rats although this hypothesis would require further experimental investigation.

Moreover, 5-HT, ET-1 and Phe are pulmonary arterial vasoconstrictors with different modes of actions. Smooth muscle contraction is well known to be both dependent and/or independent on cytosolic calcium increase depending on the vasoconstrictor. When smooth muscle contraction is calcium independant, the contraction is due to calcium sensitization of the contractile proteins. On the one hand, ET-1 and 5-HT increase intracellular calcium by acting on receptors localised in both smooth muscle and endothelial cells (namely 5-HT_2A_, 5-HT_1B/D_, ET_A _and ET_B _in PASMC and 5-HT_2B _and ET_B _in endothelial cells) whereas Phe acts on α_1_-adrenoceptors on PASMC only [14,21,30,31]. On the other hand, although 5-HT, ET-1 and Phe activate inositol 1,4,5-triphosphate-induced intracellular calcium release, 5-HT and Phe also stimulate voltage-independent calcium permeable channels (namely receptor- and/or store-operated channels) whereas ET-1 rather stimulates voltage-dependent calcium channels [32-34]. Finally, contraction to ET-1 and Phe are strongly dependent on calcium sensitization of contractile proteins whereas contraction to 5-HT is only slightly dependent on this process [21,35-37]. Altogether, such differences in the contractile mechanisms might explain why the effects of the gap junction blockers differ according to the agonist used.

Regarding receptor-independent depolarisation, we have observed a significant decrease in the contraction to KCl in IPA from MCT rats but an increase in CH rats (Figure 3D). Moreover, ^37-43^Gap27 decreased the sensitivity to high potassium solutions in IPA from CH rats only, thus inducing a contraction similar to the one observed in the N rats (Figure 7C). These results suggest that Cx 37 and/or 43 were involved in the hypersensitivity to KCl in CH rats. This result is thus in good agreement with the expected role of Cx in the conduction of the electrical activity observed in various systemic vessels [38].

## Conclusions

In conclusion, we have evidenced the presence of functional Cx 37, 40 and 43 not only in normal but also in pathological pulmonary vessels. These three Cx participate to pulmonary arterial vascular contractility in response to both receptor-dependent and receptor-independent stimuli in N, CH and MCT rats. However, the role of these Cx in the vasoreactivity of pulmonary artery varies according to the stimulus and the rat model. These results suggest that Cx may act as specific therapeutic targets for different type of PH.

## List of used abbreviations

CH: chronic hypoxia; COPD: chronic obstructive pulmonary disorder; CRC: concentration-response curve; Cx: connexins; ET-1: endothelin-1; 5-HT: serotonin; IPA: intrapulmonary arteries; IPA1: intrapulmonary artery of the first order; IPA3: intrapulmonary artery of the third order; LV+S: left ventricle plus septum; MCT: monocrotaline-treated; mean PAP: mean pulmonary arterial pressures; N: normoxic; O_2_^●^: superoxide anion; PA: pulmonary artery; PAH: pulmonary arterial hypertension; PASMC: pulmonary arterial smooth muscle cells; PH: pulmonary hypertension; Phe: phenylephrine; RV: right ventricle.

## Authors' contributions

MB, DD, RM, JPS and CG contributed to the conception and design of the study. MB, DD and CG performed experiments, evaluated results and interpreted data. MB, DD, RM, JPS and CG were involved in drafting and revising the manuscript. All authors read and approved the final manuscript.
